# The slip-and-slide algorithm: a refinement protocol for detector geometry

**DOI:** 10.1107/S1600577517013327

**Published:** 2017-10-18

**Authors:** Helen Mary Ginn, David Ian Stuart

**Affiliations:** a Division of Structural Biology, Wellcome Trust Centre for Human Genetics, Roosevelt Drive, Oxford OX3 7BN, UK; b Diamond House, Harwell Science and Innovation Campus, Fermi Avenue, Didcot OX11 0QX, UK

**Keywords:** serial crystallography, free-electron laser, SFX, stills, XFEL, detector, geometry

## Abstract

Geometry correction is traditionally plagued by mis-fitting of correlated parameters, leading to local minima which prevent further improvements. Segmented detectors pose an enhanced risk of mis-fitting: even a minor confusion of detector distance and panel separation can prevent improvement in data quality. The slip-and-slide algorithm breaks down effects of the correlated parameters and their associated target functions in a fundamental shift in the approach to the problem. Parameters are never refined against the components of the data to which they are insensitive, providing a dramatic boost in the exploitation of information from a very small number of diffraction patterns. This algorithm can be applied to exploit the adherence of the spot-finding results prior to indexing to a given lattice using unit-cell dimensions as a restraint. Alternatively, it can be applied to the predicted spot locations and the observed reflection positions after indexing from a smaller number of images. Thus, the indexing rate can be boosted by 5.8% using geometry refinement from only 125 indexed patterns or 500 unindexed patterns. In one example of cypovirus type 17 polyhedrin diffraction at the Linac Coherent Light Source, this geometry refinement reveals a detector tilt of 0.3° (resulting in a maximal *Z*-axis error of ∼0.5 mm from an average detector distance of ∼90 mm) whilst treating all panels independently. Re-indexing and integrating with updated detector geometry reduces systematic errors providing a boost in anomalous signal of sulfur atoms by 20%. Due to the refinement of decoupled parameters, this geometry method also reaches convergence.

## Abstract 2

Geometry correction is performed with separation of Euclidean and non-Euclidean movements with respect to the sample, refined against independent target functions derived from the data. This leads to substantial improvements in indexing rates and data quality indicators and refines to convergence.

## 1. Introduction   

A key part of data processing for any diffraction experiment, including X-ray diffraction, is determining the parameters of the experiment: those of the sample (*e.g.* unit-cell dimensions, crystal orientation, size, mosaicity), those of the probe (*e.g.* the beam focus, divergence, energy) and the recording device (*e.g.* detector gain curve, angle relative to beam, placement relative to sample, segment placements relative to each other). Due to well characterized detectors present in most synchrotron beamlines, researchers benefit from very good calibration of these detectors, including gain and precise knowledge of the placement of the sensors with respect to each other.

The machines with the most brilliant hard X-ray sources are now X-ray free-electron lasers (XFELs): the Linac Coherent Light Source (LCLS) and the SPring-8 Angstrom Compact Free Electron Laser (SACLA) are already in use and the European XFEL (EuXFEL) and SwissFEL have begun lasing. These are equipped with custom, in-house detectors, which embody technological advances necessary to cope with the demands of recording XFEL data, including: the Cornell-SLAC hybrid Pixel Array Detector (CSPAD) (Hart *et al.*, 2012 ▸) at LCLS, the MultiPort Charged Coupled Device (MPCCD) (Kameshima *et al.*, 2014 ▸) detector at SACLA, and the upcoming Adaptive Gain Integrating Pixel Detector (AGIPD) (Henrich *et al.*, 2011 ▸) and the Jungfrau detector (Mozzanica *et al.*, 2014 ▸) at the EuXFEL and SwissFEL, respectively. The former two detectors are widely used at their respective light sources. These detectors are all segmented, and are being upgraded frequently. The CSPAD in particular is a 64-segmented detector of application specific integrated circuit (ASIC) modules which are bump-bonded into pairs. This requires regular dismantling and rebuilding, which inevitably changes the relative positions of the detector segments. This is an important part of the technological development of these detectors. However, the frequent (and unknown) rearrangement of the detector panels has a pronounced effect on the ability to process the data. The resulting panel shifts are largely unknown, and have to be back-calculated from data collected at the XFEL. For the regular crystallographer, the careful calibration of commercial detectors at synchrotron beamlines may have allowed this aspect of detector calibration to drift to the back of the mind.

XFEL data provide the biggest challenge for back-calculation of detector geometry. At an XFEL, crystals enter the beam with an unknown orientation, and a portion of X-rays entering a crystal will satisfy the Bragg condition and diffract to produce a measurable spot on the detector. A crucial stage of the data analysis workflow is working out the relative positions of the putative reflections which gave rise to these spots. This dictates how X-rays are traced from spot to sample and determines the intersection with the Ewald sphere, a critical component of determining crystal orientation. The geometry is also essential for predicting the positions of the diffracted X-rays in preparation for integrating reflection positions. Even a sub-pixel offset in the assigned position of a panel from its true position will disturb the integrated value for multiple reflections.

Geometry refinement calculations have been carried out by a number of groups. Work in early phases of XFEL data processing noted the requirement for further detector geometry refinement from the initial parameters, being incorporated into *geoptimiser* (Yefanov *et al.*, 2015 ▸), a stand-alone software package associated with *CrystFEL* (White *et al.*, 2016 ▸), and a geometry (also termed metrology) refinement module in *cctbx.xfel* (Hattne *et al.*, 2014 ▸). Many of the authors of the latter went on to incorporate a different form of detector geometry refinement in the DIALS framework for goniometer-based experiments and this has been adapted to exploit XFEL data (Waterman *et al.*, 2016 ▸; Brewster *et al.*, 2017 ▸).

The first two XFEL-specific methods both concentrate on the challenging arrangement of the CSPAD detector. The methods of geometry refinement by Hattne *et al.* (2014 ▸) were carried out using an initial GUI-based manual adjustment of the summed-image powder rings to obtain an initial starting solution, which is performed automatically using *geoptimiser*. Both of these reported methods then refine the positions and rotations of all ASIC pairs on the CSPAD, the orientation and unit-cell dimensions for each crystal, and the beam translation and sample-to-detector distance by least-squares refinement to minimize the discrepancy between observed and predicted spot positions from known indexed lattices. On the other hand, Yefanov *et al.* (2015 ▸) refined three parameters per individual panel: translation in *X* and *Y* (axes perpendicular to the beam) and a rotation around the centre of the panel, all kept in plane with no variation in the *Z* axis. Although this appears to be a less ambitious approach, because certain combinations of parameters can look remarkably similar, refining fewer parameters prevents one parameter from compensating for errors in another correlated parameter. As a result, this method may be advantageous against the chosen target function.

Detector geometry refinement in DIALS aims to refine geometry for both single-shot experiments as per XFEL experiments and goniometer-based rotation experiments at synchrotrons. This is a more ambitious project which includes fully three-dimensional detector models with an optional hierarchical structure, potentially able to model the panels arranged on a cylinder of the Pilatus 12M detector at Diamond, beamline I23 (Wagner *et al.*, 2016 ▸). In the DIALS framework, during refinement, panels are rotated with respect to their basis vectors, and also translated along these three vectors (which are not necessarily parallel to laboratory framework axes). However, several combinations of parameters defined in this model are still correlated, which significantly complicates the process of refinement.

**Figure 1:**
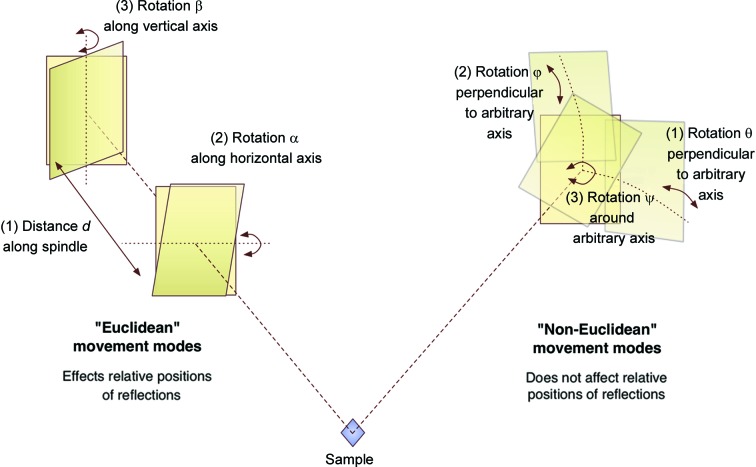
Types of movement: Euclidean (left) and non-Euclidean (right). Left: the detector panel can move backwards and forwards on the spindle connecting its midpoint to the sample by distance *d*, and rotate by two angles on two axes orthogonal to the spindle axis (α and β). This will affect the predicted spot locations (or, conversely, it will affect the relative arrangement of back-projected rays). Right: the detector panel can move around polar coordinates θ, φ and ψ around any arbitrary axis passing through the sample. This will not affect the relative arrangement of ray projections back onto the Ewald sphere, only their absolute position relative to the beam centre.

**Figure 2:**
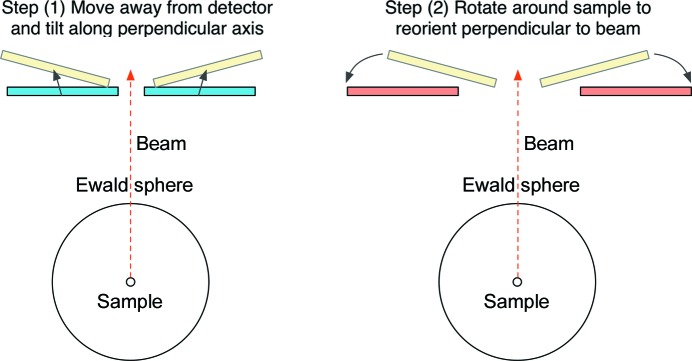
A lateral movement in panels must be split into the Euclidean elements (step 1, left) and the non-Euclidean elements (step 2, right). The former aligns the spot projections or predicted reflection positions within a single panel. The latter aligns the panels relative to the beam centre or each other.

In this paper, we present a new method of parameterization of a detector in three dimensions which aims to deconvolute the vast majority of the correlated parameters. These can be split into two major modes of movement: Euclidean movements which affect the relative arrangement of putative reflection coordinates in reciprocal space, and non-Euclidean movements of spherical geometry which rotate a panel around the sample but do not affect the relative arrangement of putative reflection coordinates (Fig. 1 ▸). This has been termed the slip-and-slide algorithm, as one first ‘slips’ into the correct orientation relative to the sample (Euclidean movements) and then ‘slides’ around the sample (non-Euclidean movements). These can be refined against different target functions. Refining these movements separately prevents a one-step movement in the *X* axis, and this must be decomposed into its component modes (Fig. 2 ▸). In addition, we present two types of target function, one of which uses the traditional approach of comparing observed to predicted pixel positions of diffracted rays through illuminated Bragg peaks, and another function which takes advantage of the ‘unit cell fingerprint’ of the lattice-derived pattern of spots on the detector, with no concern given to the orientation of the lattices.

The effect of this method of geometry refinement on even a data set of high initial quality is demonstrated by observing the effect on an independent parameter (anomalous signal from sulfur), which should indicate the relative importance of geometry calibration. This corroborates the message from a study on sulfur SAD phasing of thaumatin crystals (Nass *et al.*, 2016 ▸) which found that applying further geometry correction to gadolinium-phasing of lysozyme (Barends *et al.*, 2014 ▸) could reduce the required patterns from 60000 in the original study to 7000.

## 2. Materials and methods   

### 2.1. Data input   

The initial geometry was loaded from the *CrystFEL* geometry file for the CSPAD (at LCLS) into the *cppxfel* suite. Files in the CXI format (using the HDF5 specification) were created using *Cheetah* (Barty *et al.*, 2014 ▸) and can now be directly loaded into *cppxfel* (Ginn *et al.*, 2016*a* ▸). Cypovirus type 17 polyhedrin (CPV17) diffraction patterns from a prior study (Ginn *et al.*, 2015*b* ▸), selected using *Cheetah* hit-finding [data deposited in CXIDB (Maia, 2012 ▸), code 29], were used, from LCLS proposal LS06. Note that, for the CSPAD, the bump-bonded ASIC pairs were treated as a single unit.

### 2.2. Detector geometry model   

**Figure 3:**
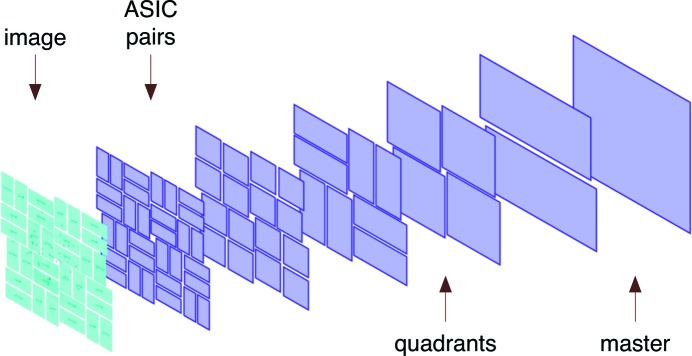
Detector hierarchy for a CSPAD image (left). Subsequent images show the pairs of detector groups joined in a higher-level group from the previous image, finishing with the final ‘master’ group. Each group will inherit the properties of the parent group recursively. This enables blanket changes to be made to the entire detector or specific sections of it, and also aids the process of refining geometry from unindexed diffraction patterns.

The underlying model of the detector geometry is no more complex than that implemented in DIALS. The laboratory basis vectors were chosen such that the *Y* axis pointed to the ceiling, the *Z* axis along the beam from sample to detector, and the *X* axis in the appropriate direction to complete a right-handed orthogonal coordinate system. The origin was defined as the sample position. Each segment of the detector stored three properties: its midpoint coordinate, and the direction vectors which specify the fast-scan and slow-scan axes (from which the orthogonal vector may be derived). The 32 individual ASICs of the detector were paired together to form 16 ASIC pairs. Individual ASICs first inherited the properties from these higher-order ASIC pairs, before applying further corrections. These 16 ASIC pairs were paired recursively, ending with the definition of a single ‘master’ detector object (Fig. 3 ▸).

During the course of refinement, small offsets are applied to the detectors in order to minimize the target function, which are applied on-the-fly. At the end of each cycle of refinement of a detector panel, these small offsets (rotation angles and translations as defined in §2.3) are absorbed into the direction vectors which specify the fast- and slow-scan axes. The small offsets are then set to zero before the next round of refinement. This is merely a detail of the implementation.

### 2.3. Panel movement modes   

Movement modes were divided into Euclidean and non-Euclidean movements. The three Euclidean movements are: (*a*) movement back and forth along a spindle connecting the centre of the panel to the sample, (*b*) a rotation around an axis normal to the spindle, and (*c*) a rotation around the other orthogonal axis normal to the spindle. There were three types of non-Euclidean movement, polar coordinates θ, φ and ψ around any axis passing through the sample (Fig. 1 ▸).

### 2.4. Refinement of unit-cell dimensions   

To refine unit-cell dimensions independently from detector geometry, a combination of over-prediction and a local search for the highest peak, followed by integration, could ‘catch’ reflections which would not satisfy the Bragg condition using the parameters of the initial model. This identified illuminated reflections which were further afield from the initial prediction, and was followed by initial orientation refinement. Reflections were registered as either strong or not strong, and thus provided data which were independent of the diffraction geometry. Global unit-cell refinement was then performed to bring the unit-cell dimensions in line with what was required to focus the reflections around the mean wavelength. Iterations of orientation refinement followed by unit cell refinement were performed until convergence.

### 2.5. Geometry refinement using indexing solutions   

Diffraction patterns of CPV17 run 4 were indexed using an initial estimate of the detector geometry. The initial offsets between the predicted and observed locations for the vast majority of ASIC pairs were within 0 to 2 pixels, which was also affected by the unit cell and distance correction described above. Indexing was performed using the TakeTwo algorithm (Ginn *et al.*, 2016*b* ▸), including solution validation. During indexing, the surrounding area up to five pixels away from the predicted peak was searched, and the highest pixel value stored as an offset. The offset was stored in both pixels and in reciprocal coordinates when back-projected onto the Ewald sphere. This local search was also used during the error checking process of indexing solutions for the initial geometry only, which relaxes the restraints. For the validation of indexing rates determined with refined geometry, the local search was not performed. Only the strongest peaks were recorded using an equivalent of an I/SIGI cutoff of 0.67 (using a detector gain of around 9.0 ADU per photons for the 1.46 Å wavelength pulses used).

The Euclidean movement modes were refined first using the Nelder–Mead algorithm (Nelder & Mead, 1965 ▸) against a measure of the spread of the pixel offsets. As there were many incorrectly located peaks, a least-squares offset would put undue emphasis on outliers where the majority of the errors are not similar to those derived from a Gaussian error model. Instead, an empirical ‘reward-based’ target function was developed (rather than penalizing for being far from the true value).

**Figure 4:**
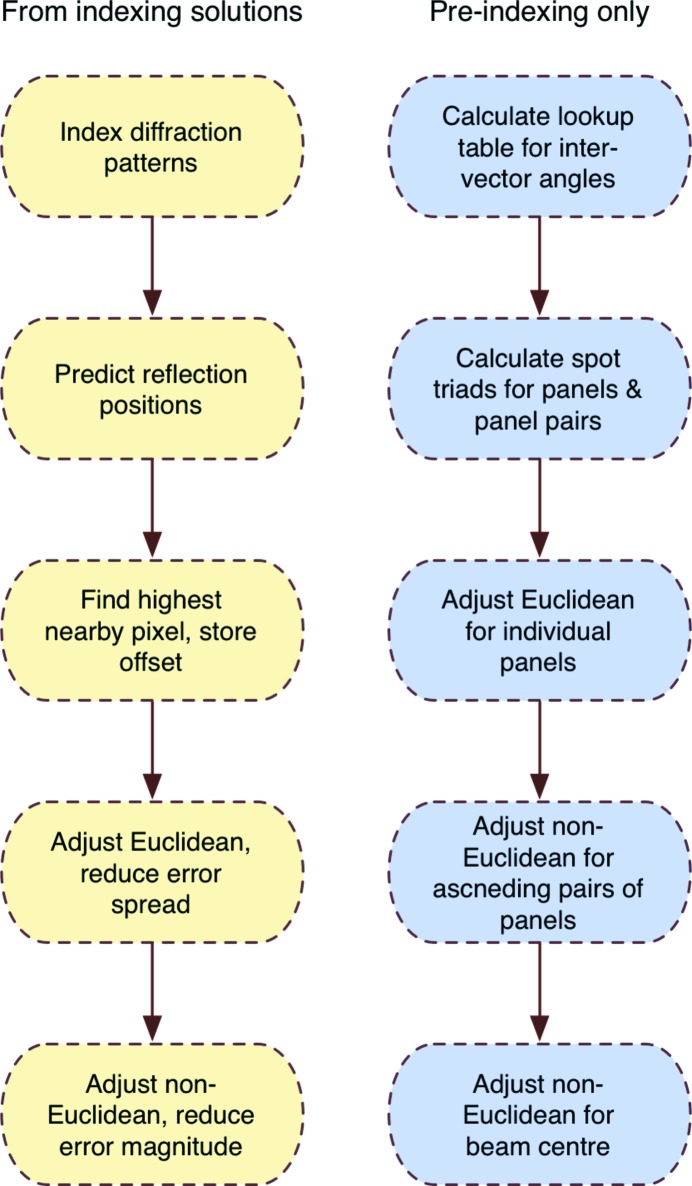
The order of events is shown for geometry refinement from indexing solutions for individual crystals (left) and for using the pre-indexing ‘lattice-like’ information only (right).

The equation E which was to be maximized using the Euclidean movement modes only is described in equation (1) below. This calculated and summed a value for every strong reflection *i*, which was the sum of an exponential function of the axis offsets *x* and *y* of every other reflection *j* from reflection *i*. Hence this was independent of the origin of the pixel offsets and would be best maximized by reducing the spread of neighbouring offsets. The non-Euclidean movements were refined against the deviation of the reciprocal offsets (in reciprocal coordinates) from the origin. When X-rays diffract through a finite reciprocal lattice point at a wide angle and hit a detector far from the perpendicular, the rays will be distributed with an increasing spread of incident angles. The choice to calculate in reciprocal offsets guarded against the over-compensation which would occur by trying to centre on the midpoint of the pixel offset distribution. This was calculated using a related equation (2) using non-Euclidean movements to rotate the panels until the offset is zero. Equation (2) is less computationally expensive than equation (1). The constant *k* in both equations is a scale factor to roughly scale the offsets to the appropriate scale, and was set to 5.0 for pixel offsets and 0.002 Å^−1^ for reciprocal coordinate offsets. The flow of operations are described in Fig. 4 ▸.
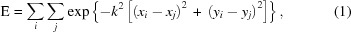


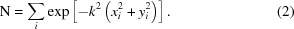
Each mode of movement was refined independently for each panel. This ensures that any global trends observed in the detector have not been biased by using a hierarchical detector. This means any trends in overall tilt or detector distance can be determined independently.

### 2.6. Geometry refinement using unit-cell information only   

Spot-to-spot vectors were determined in the same manner as previously described (Ginn *et al.*, 2016*b* ▸). ‘Intra-panel’ vectors are vectors which span two spots on the same detector panel, whereas ‘inter-panel’ vectors are those which have two spots each from a different panel.

For a given panel, all pairs of intra-panel vectors sharing a common spot were stored, and on each iteration of refinement the two reciprocal lengths of the vectors and the angle between them (restricted to 0 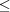 angle 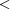 90) were recalculated. This produces a three-dimensional histogram of two lengths, and angle and a frequency. When summed over multiple still shots from several angles, a sampling of all potential vector pairs is expected. For each panel, this histogram was refined against the ideal histogram calculated from the unit-cell dimensions, using the Euclidean movement parameters. This is refined using the Nelder–Mead algorithm to convergence.

Inter-panel vectors between two chosen panels were paired either with other inter-panel vectors or intra-panel vectors on one panel or the other, as long as they shared a common spot. The three-dimensional histogram was generated and refined against the non-Euclidean movements of the two panels next to each other. The movement is defined by the polar coordinates θ, φ and ψ, the axis for which was defined as the midpoint between the centres of the two chosen panels. The panels are restricted to perform equal and opposite movements (*e.g.* rotation by θ of one panel must be opposed with a −θ rotation of the other), to prevent unnecessary drift of panels around the experimental hutch. A hierarchy of panels was formed for both the CSPAD and MPCCD detectors, with ascending pairs of panels up to the entire detector. For the CSPAD, adjacent ASIC pairs are paired, followed by paired ASIC foursomes, up to quadrants, halves and then the master detector. Each level of the hierarchy was refined using the Nelder–Mead algorithm to convergence.

The target function for the unit-cell fingerprint was calculated from the ideal unit-cell dimensions and a reciprocal-lattice-point radius (akin to the profile radius in *CrystFEL* or the crystal domain size for *cctbx.xfel*). These acted as tolerances for the lengths of vectors to be accepted as resulting from the chosen lattice parameters. Vectors were rejected if the magnitude of the vector exceeded 0.15 Å^−1^ (reciprocal coordinate units). The angles were calculated from the tolerances on the lengths of the vectors such that shorter vectors had wider tolerances. These are stored in memory with 240 voxels sampling each dimension, producing 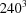 single-precision floating points (occupying 55.3 MB in memory). Each voxel contained a ‘score’ for a given combination of two vector lengths and the angle. This was a linear ascent from 0 at the edge of the acceptable tolerance to 1 at the most ideal calculated value. Sets of three spots were taken for each target function, and the three vectors between them were stored in memory. For the target function, a sum of the individual scores for each spot triad were calculated. Each pair of the three vectors in a spot triad were given a score between 0 and 1 according to the lookup table, and multiplied together, which can also be calculated in advance.

### 2.7. Calculation of anomalous signal   

Diffraction patterns were indexed using a given geometry file and orientation matrices were refined according to a previous protocol (Ginn *et al.*, 2015*b* ▸). The peak locations for these orientations were then predicted, and could be treated as exact or approximate. For the former, images were integrated at the exact peak locations; for the latter, after a three-pixel local search for the highest pixel value, on which the integration window was centred. Integration and post-refinement of these diffraction patterns were performed as previously described (Ginn *et al.*, 2015*a* ▸) and anomalous differences were calculated per cycle.

## 3. Results   

Geometry movements were categorized into either ‘non-Euclidean’ or ‘Euclidean’ movements. The non-Euclidean geometry movements are those which do not affect the relative positions of diffracted rays on the surface of the panel, *i.e.* any movement on a sphere around the sample of a constant radius. The other movements are those which only affect the relative positions of the diffracted rays and are classed as Euclidean movements. These two modes of movements are refined against separate target functions, involving no component to which the parameters are insensitive. This means that, apart from the exception of moving an entire single-panel detector forwards or backwards, any single movement along the traditional *X*/*Y*/*Z* axes is disallowed. These movements must be decomposed into the Euclidean and non-Euclidean elements (Fig. 2 ▸) and refined against separate target functions.

Two methods can be used to refine the geometry, depending on whether indexing solutions have been obtained or not. If the geometry is far from the truth, orientation matrices may be extremely difficult to determine. However, the images contain a great deal of information as the spots still obey a ‘lattice-like nature’ defined by the unit-cell parameters and space group centring. Hence the Euclidean and non-Euclidean movements can be refined against related target functions, depending on whether the geometry is close to the true positions or needs to be refined from a far starting position.

### 3.1. Corrections to unit-cell parameters   

The unit-cell dimensions and detector geometry parameters must be carefully separated. Using the same target functions to refine the unit-cell dimension as to refine the detector geometry will lead to incorrectly determined parameters. Hence, a method to abstract the information necessary for unit-cell refinement from the detector geometry is performed instead (see §2.4). This clusters the prediction of the strong reflections around the mean wavelength recorded by integration of the spectrometer output at the XFEL for both data sets. This method was used to update the unit-cell dimensions used for CPV17 from 106.1 Å to 105.5 Å (deflation of approximately 0.6%). An approximate correction was applied to the initial detector distance (inflation by 0.6%) to compensate for the change in unit cell.

### 3.2. Indexing-driven geometry refinement   

**Figure 5:**
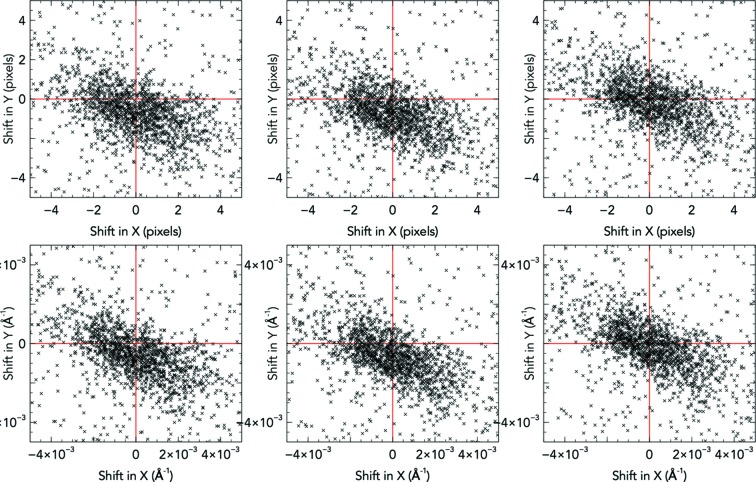
The shifts in predicted reflection positions on a mid-resolution panel at the start (left), after Euclidean movements (centre), and after non-Euclidean movements (right). The former is refined against the spread of pixels (top row), resulting in a reduction of the standard deviation of reciprocal offsets by 0.8% (including outliers), whereas the latter is refined against the centre-point of the reciprocal offsets (bottom row), resulting in a reduction of the mean reciprocal offset by 77.1%.

**Figure 6:**
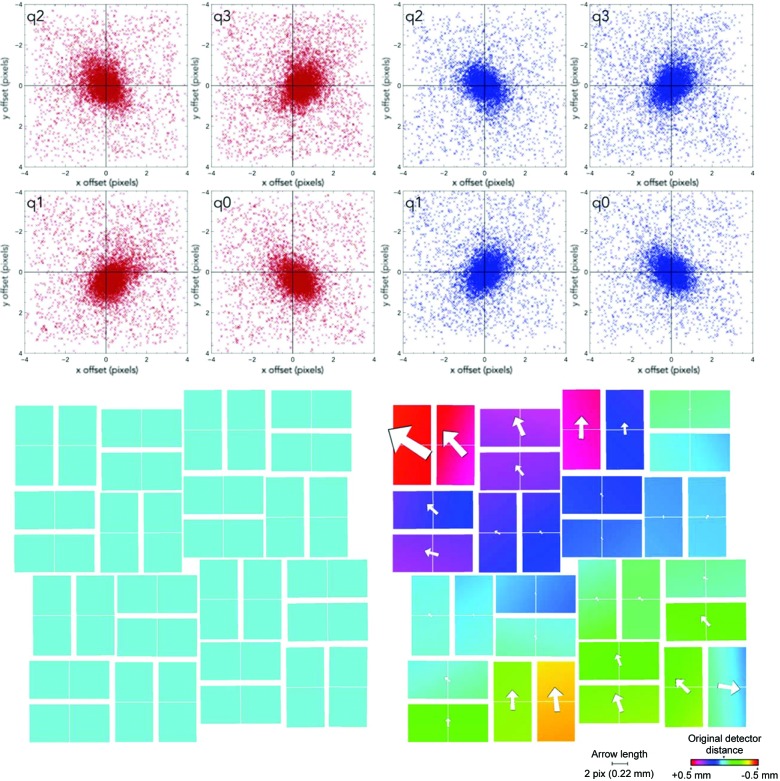
The shifts of the pixel offsets from the predicted and observed peak locations in each quadrant of the CSPAD from the original offsets in the geometry file (top, red), and after refinement (top, blue). On the bottom is the before and after CSPAD corresponding to the graphs above, with the *Z* axis spanning a 1 mm rainbow around the original detector distance. Arrows shown are proportional to the offset in *X* and *Y* of each panel from the original position and independent of the *Z* axis. Geometry refinement performed using 170 indexing solutions.

If the detector geometry is sufficient to index and produce pixel offsets not diverging too far from zero, the Euclidean and non-Euclidean movements can be refined against the appropriately sensitive target functions. The Euclidean movements are refined against the spread of pixel offsets (but need not be equal to zero), and the non-Euclidean movements are refined against the spread of reciprocal coordinate offsets being centred around zero. The result of applying the two-stage protocol on individual panels is presented in Fig. 5 ▸, and the overall effect on the pixel offsets on each quadrant is shown in Fig. 6 ▸.

The TakeTwo algorithm (Ginn *et al.*, 2016*b* ▸) may be able to index with very poor geometry, in the case where the independent panels are well oriented in their Euclidean movements but are poorly oriented with respect to either each other or the beam centre. If there are enough panels oriented correctly with respect to the sample, and enough inter-connected panels to find enough vectors consistent with a single indexing solution, identifying the correct basis vectors is very likely to be successful. However, the pixel offsets may be extremely divergent. If the indexing rate is very low to begin with, pre-indexing geometry refinement can be performed to help bootstrap indexing.

### 3.3. Pre-indexing geometry refinement   

**Figure 7:**
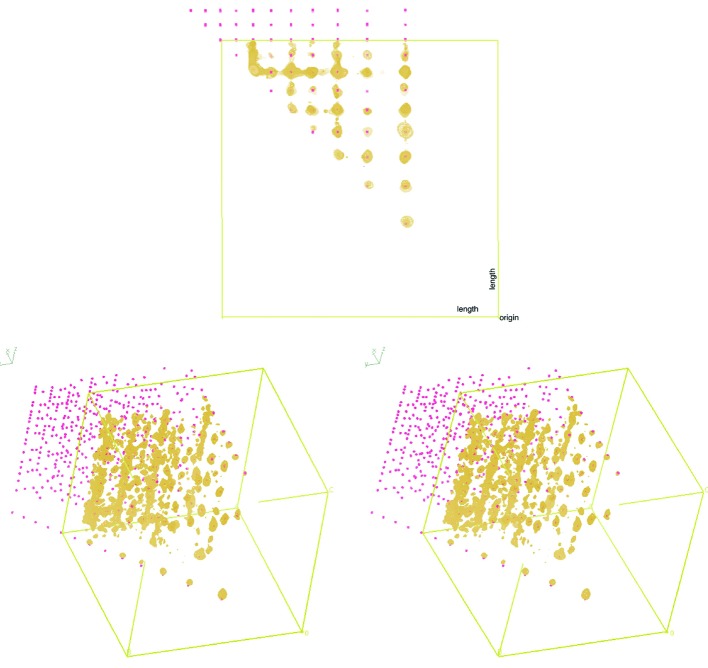
Representation of the three-dimensional histogram of inter-spot vectors using *Coot*. Atoms denote the positions of the expected positions, calculated with a buffer region outside of the observed vector positions (within the bounds of the unit cell). The map density reflects the set of observed vectors, contoured at 6.5σ. Reciprocal distances cover the range 0–0.04 Å^−1^. The top diagram is shown with all angles projected down perpendicular to the page. The bottom diagram is a stereo image showing two lengths (*x*, *y* axes) and angle (*z* axis) illustrating all dimensions.

By analysing pairs of spot-to-spot vectors, one can essentially produce an orientation-less ‘unit-cell fingerprint’ of the space group and centring (disregarding axial systematic absences). This unit-cell fingerprint will be perturbed if the non-Euclidean movement parameters of a single panel are incorrect, and so can be used as a target function to lock this information. This is demonstrated graphically in Fig. 7 ▸.

Due to the lack of exact orientation information, it is harder to lock panels in an absolute position relative to the beam centre. However, it is possible to lock panels to one another, starting with pair-wise interactions between independent panels, and then pair-wise interactions between pairs, in an ascending hierarchy, until the whole of the detector has been aligned with itself. The inter-panel vectors contain the information which defines the relative positions of the two participating panels and this forms the basis of this part of the calculation.

Finally, the entire detector must be aligned with the beam centre. However, this is unlikely to diverge far from the original centre, since locking onto an alternative Miller index is likely if only the entire tilt of the detector has exceeded the 2θ scattering angle of the lowest resolution Bragg peak (in the case of the CPV17 unit cell, this is 1.1°).

### 3.4. Geometry refinement reveals a tilt in the CSPAD   

**Figure 8:**
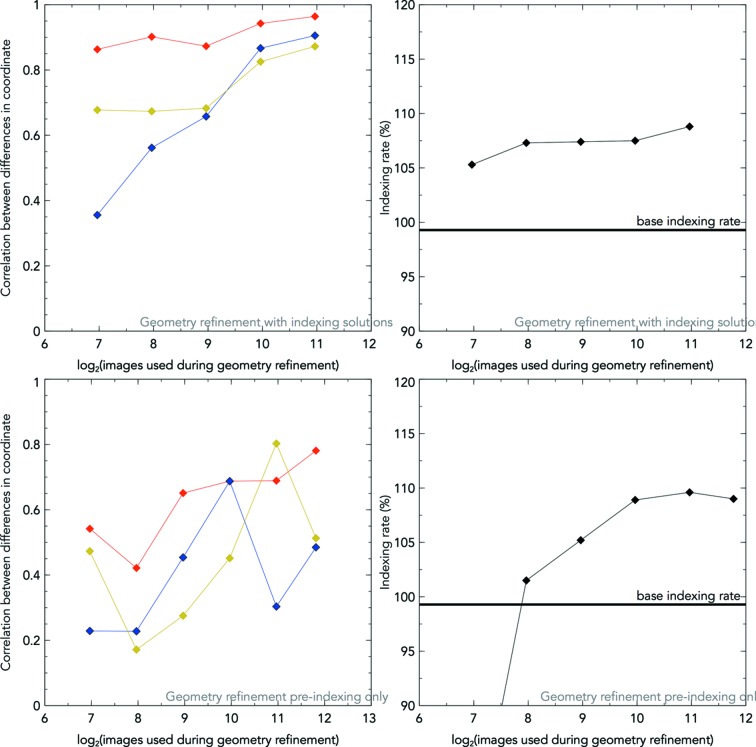
From the CPV17 data set: the correlation coefficients of the *X* (blue), *Y* (yellow) and *Z* (red) coordinate offsets of the four panel corners from the original positions are plotted (left), from 125 images to 2000 images (top, with indexing solutions) and from 125 to 3500 images (bottom, pre-indexing only). At the top right, the base indexing rate is shown in black for the original, partially refined *CrystFEL* format geometry file for CPV17, with a local search size of 5 pixels for the maximum peak (used for indexing solution verification). After refinement from indexing solutions, with a local search size reduced to 0 in all cases, the improvement in the indexing rates are shown. A noticeable improvement in the indexing rate can be seen from 125 images using indexing solutions. At the bottom right, 125 images fail to produce verifiable indexing solutions, but 250 images produce a small improvement in the indexing rate.

Geometry refinement using the indexing solutions produces a 5.8% boost in indexing rate using information from only 125 images of CPV17 data used for a previous structure solution (Ginn *et al.*, 2015*b* ▸). The boost in the indexing rate is comparable with that using 500 unindexed patterns (Fig. 8 ▸). The former method of refinement can exploit more information and therefore reaches convergence faster than using pre-indexing information only. However, the calculation time scales proportionally to the square of the images from indexing solutions [due to equation (1)], but is directly proportional to the number of images from the pre-indexing information. So including more data has a reduced impact on the calculation time using pre-indexing information. Furthermore, the latter method cuts out the time required to index beforehand. Therefore, under time-constrained conditions, one can approach an approximate solution from a larger number of unindexed diffraction patterns, and fine-tune the geometry using a smaller number of indexing solutions.

**Figure 9:**
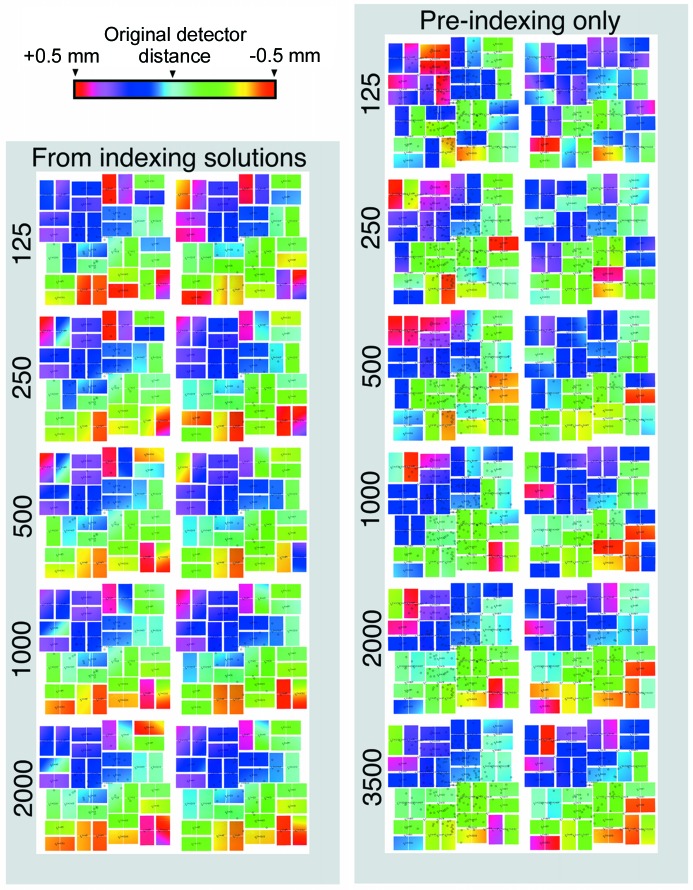
Side-by-side comparison of geometry refinement with indexed and unindexed diffraction patterns. Images of the CSPAD after geometry refinement from oriented indexing solutions (left), and using the pre-indexing information contained within the diffraction patterns only (right). Each column is divided into two half-data sets which were used to run the geometry calculation independently. The number of images which were given to each half are displayed on the left-hand side.

Because the geometry is run separately on individual panels using indexing solutions (and for the Euclidean stages of pre-indexing information), any global trends which are observed throughout the detector can be considered to have been reached in an unbiased manner. In this case, both forms of geometry refinement reveal a tilt in the detector (Fig. 9 ▸) of approximately 0.3°, which produces a 1.1% error in the *Z* axis (a detector distance in the centre of 90.3 mm). Integration and post-refinement confirms that the nominal beam centre passes through the (0, 0, 0) Miller index.

### 3.5. Improvement in random error reduction and indexing rates   

In order to judge the reduction in random error, images in the data sets were split into odd and even chunks. Indexing solutions from each half were used to direct geometry refinement using both indexing solutions and pre-indexing information only. The changes in the *X*, *Y* and *Z* coordinates of the four panel corners (all of which are a net effect of both movement modes) were stored for each half after refinement, and the correlation between them measured for increasing numbers of images. This increases with use of additional images and rises faster for indexing information than pre-indexing information (Fig. 8 ▸).

**Figure 10:**
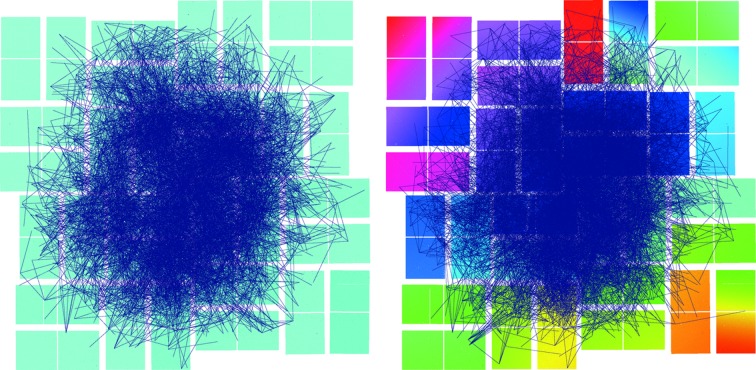
All inter-spot vectors used for indexing displayed on a single ‘iron filing’ plot, before (left) and after (right) geometry refinement with indexing solutions from 2000 images. Notice that the quadrant in the top left of the image has a poor share of the total used vectors, and, after being shifted significantly further back after refinement, has regained a more equal share of the used vectors.

The indexing rates show an immediate improvement after geometry refinement even with the minimum number of images (125 images), despite reducing the local search area to zero pixels, which produce more stringent indexing solution checks. The vectors used for indexing *via* the TakeTwo algorithm can also be plotted on a single image and this may reveal portions of the detector (or vectors between parts of the detector) which are not used during indexing. This ‘iron filing’ plot can highlight poor geometry between two areas or with an area of the detector (Fig. 10 ▸).

### 3.6. Data set quality improvement   

In order to judge the reduction of systematic error, the anomalous signal was chosen as an independent metric, using *ANODE* (Thorn & Sheldrick, 2011 ▸) to calculate the average sulfur signal from Met and Cys residues after refinement. This was performed for (*a*) the initial geometry without a local maximum search, (*b*) the initial geometry with a three-pixel maximum search, and (*c*) the best diffraction geometry without a local maximum search. Post-refinement was performed on indexing solutions derived and initially refined with the best geometry, and then post-refined with either initial or refined geometry definitions. Indexing solutions were initially obtained and refined using the best geometry, resulting in 7613 indexed lattices (from 7000 images).

**Figure 11:**
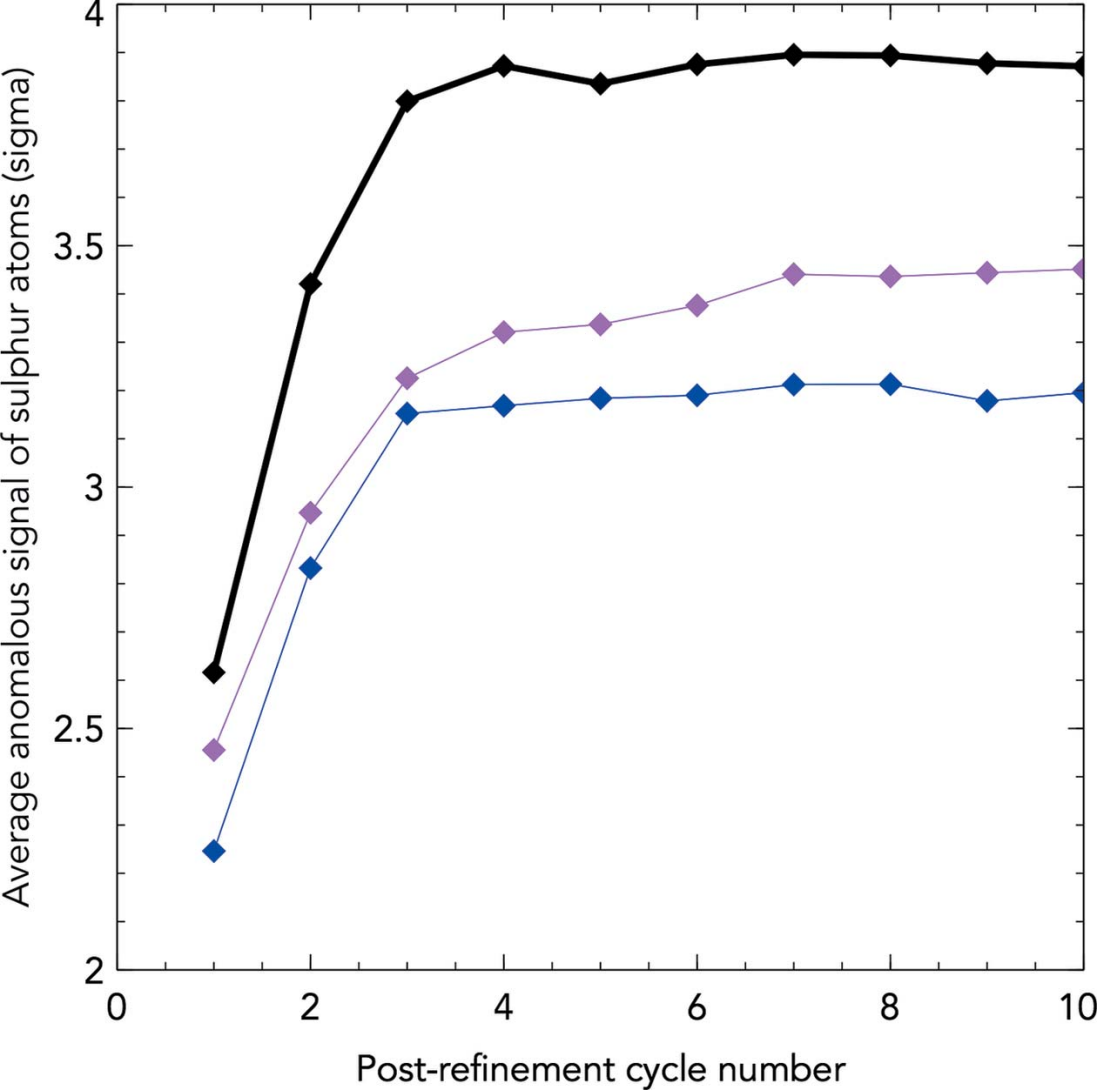
Graph showing the average sulfur atom anomalous signal per post-refinement cycle number for various treatments. 7613 orientations were derived from indexing and initial refinement using the best geometry file (2000 images and their indexing solutions). Crystal orientations were used to re-predict peak locations using the initial geometry integrating at the exact location (blue), or with a three-pixel local maximum search (magenta), or using the best geometry file and integrating at the exact location (black).

All data sets respond to post-refinement, increasing the anomalous signal per cycle (Fig. 11 ▸). Integrating the 7613-lattice data set using the initial geometry without performing any local search produced the worst anomalous signal, reaching a maximum of 3.21σ across all sulfur atoms (an increase of 43% from cycle 0). Integrating using initial geometry combined with a local maximum search stabilizes the post-refinement process, with a maximum anomalous signal of 3.45σ (an increase of 41% from cycle 0). However, this will be held back by poorly estimated integration windows for weak reflections and would be unsuitable for careful structural analysis. This causes a systematic inflation of weak reflections which perturbs the twinning H-test by skewing the intensity distribution [*Truncate* (Winn *et al.*, 2011 ▸) reports a twinning fraction of 9.1% for the reflection intensities after a local search, *versus* 0% for reflection intensities without]. The systematic inflation of weak reflections could leave a structure prone to model bias, as phases of inflated weak reflections can be manipulated to add Fourier waves supporting any correct or incorrect input model in the electron density map. Using the best geometry to predict exact peak locations for the 7613-lattice data set, the anomalous signal reaches a maximum of 3.89 (an increase of 49% from cycle 0), showing a significant improvement derived from a more accurate prediction of the spot locations, and does not systematically inflate weak reflections.

In summary, a local search for strong reflections can mitigate the effect of poor geometry definition, improving the anomalous signal over predicting at exact locations. However, it is no substitute for accurate geometry determination.

## 4. Discussion   

Using non-indexed diffraction information relies on good spot-finding information and consistent unit-cell parameters from crystal to crystal, and computation time scales with the number of images.

Using indexed diffraction information relies heavily on the ability to index, and computation time scales with the square of the number of images. However, it will be more robust for weak data where the knowledge of the predicted peak location can be used to select high value pixels that are more likely to be from a Bragg peak-diffracted X-ray. The geometry refinement described here allowed the structure solution of bovine enterovirus type 2 by using only 352 indexed diffraction patterns (Roedig *et al.*, 2017 ▸). In that case geometry refinement revealed a 0.5 mm tilt across the detector, correction for which enabled significantly more accurate prediction of Bragg spot locations and hence more reliable intensity estimates.

This algorithm leaves one type of parameter correlation not particularly well separated: the radial distance often has quite a similar effect to tilting along both of the perpendicular axes simultaneously. These effects can only be separated if there is a sufficient distortion in the spread of spots due to a tilt: the spots at one extreme end of a panel would be clustered together compared with those at the other extreme end. If the spread of angles between diffracted rays allows sufficient sensitivity to separate these effects, the geometry algorithm is capable of doing so. However, this becomes harder to separate as the wavelength becomes shorter (leading to a flatter Ewald sphere) and when the detector distance is large. This is a loss of information which is inherent to all geometry refinement experiments, and therefore experiments to acquire good geometry should be performed with a long wavelength and short sample-to-detector distance.

Because the DIALS framework adjusts the position and rotation of the detector panels with respect to the vectors which define the panel edges and the cross product of the two, the refinement of the detector at the I23 beamline would heavily emulate the non-Euclidean movement modes described here, as the panels are deliberately arranged in a circle to match the origin of the Ewald sphere (sample position). However, in the vast majority of experiments the detector panels lie on a largely flat surface.

Assuming correct unit-cell information, one can evaluate the success of the detector geometry refinement by (*a*) starting from different detector distances and seeing its ability to converge on a solution from different initial positions, (*b*) comparing the similarity of two half-data sets and their convergence and (*c*) checking that the spread in pixel or reciprocal offsets has reduced from indexing solutions. If the unit-cell dimensions are inflated or contracted from their true value, this may cause the detector to become concave or convex to try to correct for the error in the Ewald sphere curvature. Hence, the unit-cell dimensions may need correction beforehand.

This algorithm provides a method to quickly uncover previously unnoticed tilts in the detector using a very small number of images. This is achieved by taking the set of observations and dividing the target function into parts which are sensitive to different types of motions of the detector. These two parts of the target function can converge independently. Using indexing solutions, tilts revealed across the entire detector are unbiased because the hierarchy of the detector is not considered, and each panel is refined independently. The effect of geometry refinement has a positive effect on indexing solutions and anomalous peak heights. The code has been released for use in *cppxfel* (Ginn *et al.*, 2016*a* ▸).
